# Evaluation of patient management of (radio-)chemotherapy-caused mucositis with the goal of enhancing patient treatment

**DOI:** 10.1007/s00432-025-06238-2

**Published:** 2025-07-10

**Authors:** Helena Wolff, Bijan Zomorodbakhsch, Martin Schnizer, Christian Keinki, Jutta Hübner

**Affiliations:** 1 Medical Clinic II, Haematology and Internal Oncology, Jena University Hospital, Friedrich-Schiller-University, Jena, Germany; 2 Haematology and Internal Oncology, MVZ Onkologische Kooperation Harz GbR, Kösliner Straße 14, Goslar, 38642 Germany

**Keywords:** Oral mucositis, Chemotherapy, Radiochemotherapy, Patient care, Communication

## Abstract

**Purpose:**

OM is a very relevant and sometimes therapy-limiting side effect of CT/RCT. There are prophylactic and therapeutic measures available that should be recommended to all patients. This study investigated how patients were informed about oral mucositis (OM) as possible side effect of CT/RCT, how well they knew about available prophylactic and therapeutic measures from the clinical guidelines and to what extent they applied these.

**Methods:**

A standardized questionnaire on information and usage of prophylactic and therapeutic measures and patient-relevant outcomes based on the German S3 Guideline was distributed among patients in German cancer departments.

**Results:**

Only 61.6% of 114 patients were informed about OM as possible side effect by their physician and 53.2% had complaints caused by OM. An insufficient number of patients were recommended to apply prophylactic and therapeutic measures. 63.5% of the patients felt well-informed about treatment options. The most frequently recommended measure was mouth rinse (50%). Only 17.6% of patients were advised to visit a dentist.

**Conclusions:**

The measures proposed in the German S3 guideline were insufficiently recommended. To improve patient education and the quality of care, more intensive use should be made of information flyers, training of nursing staff and greater interdisciplinary cooperation. If treatment-associated OM is to be expected, dental consultations should be firmly integrated into treatment planning.

**Supplementary Information:**

The online version contains supplementary material available at 10.1007/s00432-025-06238-2.

## Introduction

Oral mucositis (OM) is a common side effect of chemo- (CT) and radiochemotherapy (RCT), affecting 20–40% of the patients who receive CT, 80% who receive high-dose CT, and almost 100% of the patients who receive RCT (Trotti, Bellm et al. 2003, Naidu, Ramana et al. 2004, Sonis 2011, Lalla, Bowen et al. 2014, Thomson M. et al. 2015, Cidon 2018, Daugėlaitė, Užkuraitytė et al. 2019, Pulito, Cristaudo et al. 2020, Al-Rudayni, Gopinath et al. 2021, Bolton 2021, Colella, Boschetti et al. 2023, Atwiine, Kyomya et al. 2024, Elsehrawy, Ibrahim et al. 2024). Risk factors include therapy-related factors and patient-specific ones: being female, having substandard oral hygiene, experiencing xerostomia, having a genetic predisposition or having impaired kidney or liver function, as well as having a history of previous tumor therapy (Bellm, Cunningham et al. 2002, Avritscher, Cooksley et al. 2004, Redding 2005, Sonis 2011, Al-Dasooqi, Sonis et al. 2013, Sonis 2013, Leitlinienprogramm Onkologie (Deutsche Krebsgesellschaft 2020, Pulito, Cristaudo et al. 2020, Danwiek, Amtha et al. 2023, Atwiine, Kyomya et al. 2024, Padure, Horhat et al. 2024). OM can develop 5–14 days after the start of tumor therapy (Raber-Durlacher, Weijl et al. 2000, Redding 2005, Epstein, Thariat et al. 2012, Al-Dasooqi, Sonis et al. 2013, Colella, Boschetti et al. 2023). It can affect patients to various degrees depending on the stage, particularly in areas such as drinking, eating, speaking, swallowing, and sleeping. Patients may be affected so severely that oral intake of food, fluids, and tumor medication is no longer or only to a limited extent possible leading to hospitalization and discontinuation of therapy (Dodd, Miaskowski et al. 1999, Bellm, Cunningham et al. 2002, Elting, Cooksley et al. 2003, Redding 2005, Al-Dasooqi, Sonis et al. 2013, Peterson, Boers-Doets et al. 2015, Kusiak, Jereczek-Fossa et al. 2020, Colella, Boschetti et al. 2023, Atwiine, Kyomya et al. 2024, Elsehrawy, Ibrahim et al. 2024, Padure, Horhat et al. 2024). Not only does this increase morbidity and mortality, it also severely inflicts the quality of life of affected patients and can lead to disruption of therapy (Martins, Borges et al. 2022, Colella, Boschetti et al. 2023, Atwiine, Kyomya et al. 2024, Elsehrawy, Ibrahim et al. 2024). Therefore, OM and its clinical management are of great interest.

Many prophylactic or therapeutic measures for OM are being discussed (Cidon 2018, Daugėlaitė, Užkuraitytė et al. 2019, López-González, García-Quintanilla et al. 2021, Colella, Boschetti et al. 2023). However, there is no reliable proof of success for any of them, and mainly palliative measures have been established (Bellm, Cunningham et al. 2002, Spielberger, Stiff et al. 2004, Redding 2005, Alterio, Jereczek-Fossa et al. 2007, Volpato, Silva et al. 2007, Pulito, Cristaudo et al. 2020, Peng, Tsai et al. 2022, Colella, Boschetti et al. 2023, Elsehrawy, Ibrahim et al. 2024). In Germany, there is a guideline that contains evidence-based measures for the treatment and prevention of OM in cancer patients (Leitlinienprogramm Onkologie (Deutsche Krebsgesellschaft 2020). It also lists various dental procedures that dentists can implement to improve oral health in cancer patients (Djuric, Hillier-Kolarov et al. 2006, Alterio, Jereczek-Fossa et al. 2007). The patients must be informed and educated about the measures to implement them sufficiently. This can increase the chance of improvement and avert the interruption of CT/RCT or hospitalization, as well as improve the quality of life. Also, it is important to recognize OM as early as possible so that appropriate measures can be taken as quickly as possible. In many cases, patients are poorly informed about potential prophylactic and therapeutic measures to prevent or treat OM. On the one hand, patients might receive a lack of information about OM from their primary caregiver (Peterson, Bensadoun et al. 2011). On the other hand, the caregivers themselves might also be ill-informed about the relevance of the disease.

A good physician-patient communication is of great interest because unnecessary cases of OM could potentially be prevented. By using a recording scale like the Oral Mucositis Daily Questionnaire (OMDQ) or observing their oral mucosa themselves, patients can detect OM earlier (Bellm et al. 2002; Stiff et al. 2006). In this study we want to find out how the communication between the physician and the patient is related to OM. The aim of this study is to clarify to what extent the patient’s physician notified them about OM as potential side effect of CT/RCT. Moreover, the purpose is to identify which therapeutic and prophylactic options the patients were informed about and which they ultimately used.

## Materials and methods

### Design

To address the topic “OM induced by CT/RCT”, a standardized questionnaire (eSupplement material e1).

was designed. We based our questions on the measures and recommendations from the German S3 Guideline (Leitlinienprogramm Onkologie (Deutsche Krebsgesellschaft 2020). The first version was tested between June and July 2022 in paper form on a group of cancer patients attending a rehabilitation program in Germany. Afterwards, it was shortened. Data from the remaining questions was used. The final version was distributed in several German cancer facilities in different parts of Germany. The questionnaires were sent to five hospitals for acute cancer care and one rehabilitation clinic with the goal of at least 100 participants. As only limited preliminary information on the specific research question was available, no sample calculation was carried out. The aim was to obtain initial indications of possible correlations for which no reliable assumptions about effect sizes were available.

### Participants

Inclusion criteria were the knowledge of the German or English language. The patients had to undergo CT/RCT. Exclusion criteria were patients who neither received the described medication nor had the possibility to fill in the questionnaire.

### Questionnaire

The questionnaire was divided into 8 sections:

1. Demographic data (one closed question: gender, one open question: age).
2. Medications (two closed questions: name of medication and optional medication, current status of cancer treatment).
3. Knowledge about oral mucositis (three closed questions: when and by whom informed about the side effect “oral mucositis”, by whom diagnosed with oral mucositis).
4. Risk factors (one closed question: risk factors of oral mucositis).
5. Communication (three closed questions: structured questions on information by the physician on treatment and information on pain treatment presented with several subquestions and a question on quality of therapy information).
6. Dentist’s procedure (two closed questions: referral to the dentist, dentist’s procedure).
7. Severity of OM (three closed questions: complaints from oral mucositis, pain from oral mucositis and limitation due to complaints).
8. Treatment of OM (one closed question: type of therapy which was performed and one open question: additive type of therapy).

The content of the questionnaire was based on the German “S3-Guideline for supportive therapy of oncologic patients” (Leitlinienprogramm Onkologie (Deutsche Krebsgesellschaft 2020)). We derived the possible answers for “risk factors” from the guideline from Sect. 7.1.2. “patient-related risk for oral mucositis”. The item “poor oral health and hygiene” was made more specific and divided in tartar, bleeding gums, bad breath and/or cavities. The answer options for items “prophylactic” and “therapeutic measures”, measured in the Likert scales, were adopted from the guidelines under Sect. 7.3. and 7.4. and rephrased in a patient-friendly way. The question about “preventive measures taken by the dentist” was adapted by the last section of 7.3.1.1 “standardized oral hygiene” in the guideline. Also “sequels of oral mucositis in daily life” were taken from the OMDQ described in the guideline (Leitlinienprogramm Onkologie (Deutsche Krebsgesellschaft 2020)) to get a comprehensive list.

Mainly closed questions were used, where the patients could choose the answers from multiple choices like lists (some questions only with “yes” or “no” as possible answers, some others with multiple choices listed). For some questions there was the possibility to add free text. Four of the closed questions used 3- or 4-point Likert scales with items for consent or dissent (In the 3-point Likert scales the participants got the possibility to choose between “Yes”, “No” or “I don’t know“ and in the 4-point Likert scale they got to choose between “highly”, “moderately”, “hardly”, and “not limited at all”). The English version of the questionnaire is available as Supplementary material.

### Statistics

All data were transferred to a statistics program (IBM SPSS 28) and frequencies were calculated. Associations were calculated using Chi-Quadrat-Tests and Monte Carlo simulations with *p* < 0.05 being considered as statistically significant.

### Ethical vote

The ethical vote was given by the Friedrich Schiller University of Jena on the 27th of July 2022 in accordance with the Declaration of Helsinki. (Reg.-Nr.: 2022-2714-Bef)

## Results

### Demographic data

A total of 122 participants could be included. 53 (43.4%) were male and 67 (54.9%) female. The mean age was 62. The youngest participant was 34 years old and the oldest was 86 years old. At the time of the survey, most of the 122 recipients (87.7%, *n* = 107) declared their treatment to be ongoing. (Table 1)

**Table 1:** Demographic data (*N* = 122)

Demographic Data	Number of participants	%
**Gender**	122	
Male	53	43.4
Female	67	54.9
Divers	0	0
No answer	2	1.6
**Age**	122	
Under the age of 50	16	13.1
50–60	37	30.3
61–70	33	27
71–80	28	23
Over the age of 80	6	4.9
No answer	2	1.6
**Tumor therapy status**	122	
Not started yet	4	3.3
Ongoing	107	87.7
Completed	11	9
**Tumor medication**	111	
Infusions with CT	101	
Infusions with other tumor drugs	14	
Pills	15	
RCT	3	

### Complaints and pain

Approximately half of the patients (53.2%, 58 of 109) declared they have or previously had complaints due to OM whereas 51 (46.8%) stated they had no complaints. To specify, most of the participants said they felt restricted when eating due to OM. Of the 58 patients who declared to have complaints due to OM, 40 (69%) also stated that they were feeling pain. Female participants were more likely to report discomfort (63%) than male participants (30%) (*p* = 0.004). This statistical significance was confirmed by Monte Carlo simulation (*p* = 0.012, 95% CI [0.010–0.014]) Also, women reported more often pain (*p* = 0.025; Monte Carlo simulation (*p* = 0.013, 95% CI [0.011–0.016])). In contrast, there was no statistically significant association between older age and discomfort or pain.

### Risk factors

We asked the patients if they had the risk factors of OM listed in the guideline. 28 of 80 (35%) patients reported to frequently have calculus, bleeding gums, bad breath, and/or cavities. 41 (51.3%) stated to often have a very dry mouth or low salivation, 3 (3.75%) that in their family gingivitis is common, 12 (15%) that they were known to have impaired kidney or liver function, and 26 (32.5%) that they have had tumor therapy before. 37 (46.3%) patients said they had none of the listed risk factors. There was no statistically significant association between the complaints and risk factors (*p* > 0.05).

### Dental care

Only 19 of 108 patients (17.6%) stated that they were recommended to visit a dentist by their treating physician. The majority with 82.4% (*n* = 89) was not. Of the 89 participants who said that they were not sent to a dentist, 40 (44.9%) stated that they went to a dentist, nonetheless. From these 40, 35% (*n* = 14) declared that no measures were taken. Therefore, nearly half of the respondents (45.4%, 49 of 108) said that they never went to a dentist during or before their therapy.

Of the 44 participants who stated that they had been to the dentist, most had their teeth cleansed (25 of 44 (56.8%)). The second and third most common treatments were oral hygiene instructions (17 of 44 (38.6%)) and root canal treatments or removals (34.1%, 15 of 44). Treatments of mucosal defects/wounds accounted for the smallest proportion (2.3%, 1 of 44). (eSupplement Fig e2)

### Medical consultation

#### Sources of information

Most of the patients (61.6%, 93 of 114) declared to have learned about the risk of developing OM as a possible side effect of their tumor therapy through their treating physician. Some of the participants gave multiple answers. Therefore, 3 (2.6%) said they got informed by their general practitioner, 9 (7.9%) by their dentist, one through the internet and hospital staff, 11 (9.6%) through other people affected by it, 4 (3.5%) through friends/acquaintances and 10 (8.8%) said they informed themselves about OM.

#### Time of information

69 out of 115 (60%) patients stated they had learned about OM as possible side effect before the start of therapy, 21 out of 115 (18.3%) when they started therapy and 4 (3.5%) only after the end of therapy. 21 (18.3%) of the patients said they were being told only during therapy.

The majority (57 of 64 (89%)) declared to have diagnosed OM themselves.

#### Satisfaction with information on treatment options

**Fig. 1:**
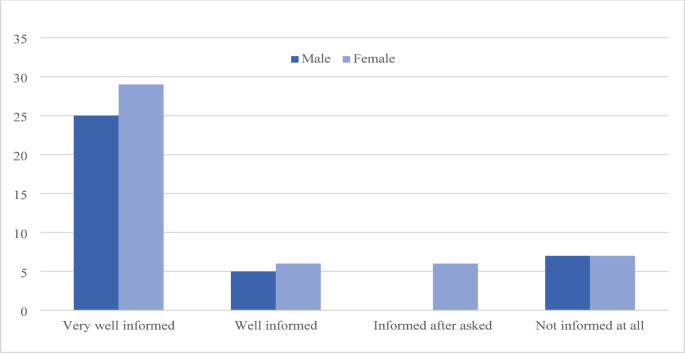
Satisfaction with information on treatment options for OM received by the treating physician (*N* = 85)

Most of the participants felt well informed by their treating physician about treatment options: 54 of 85 of the participants (63.5%) declared that their physician recommended prophylactic and therapy options and explained them comprehensively. (Fig. 1)

### Adherence to methods to prevent or treat OM

**Fig. 2:**
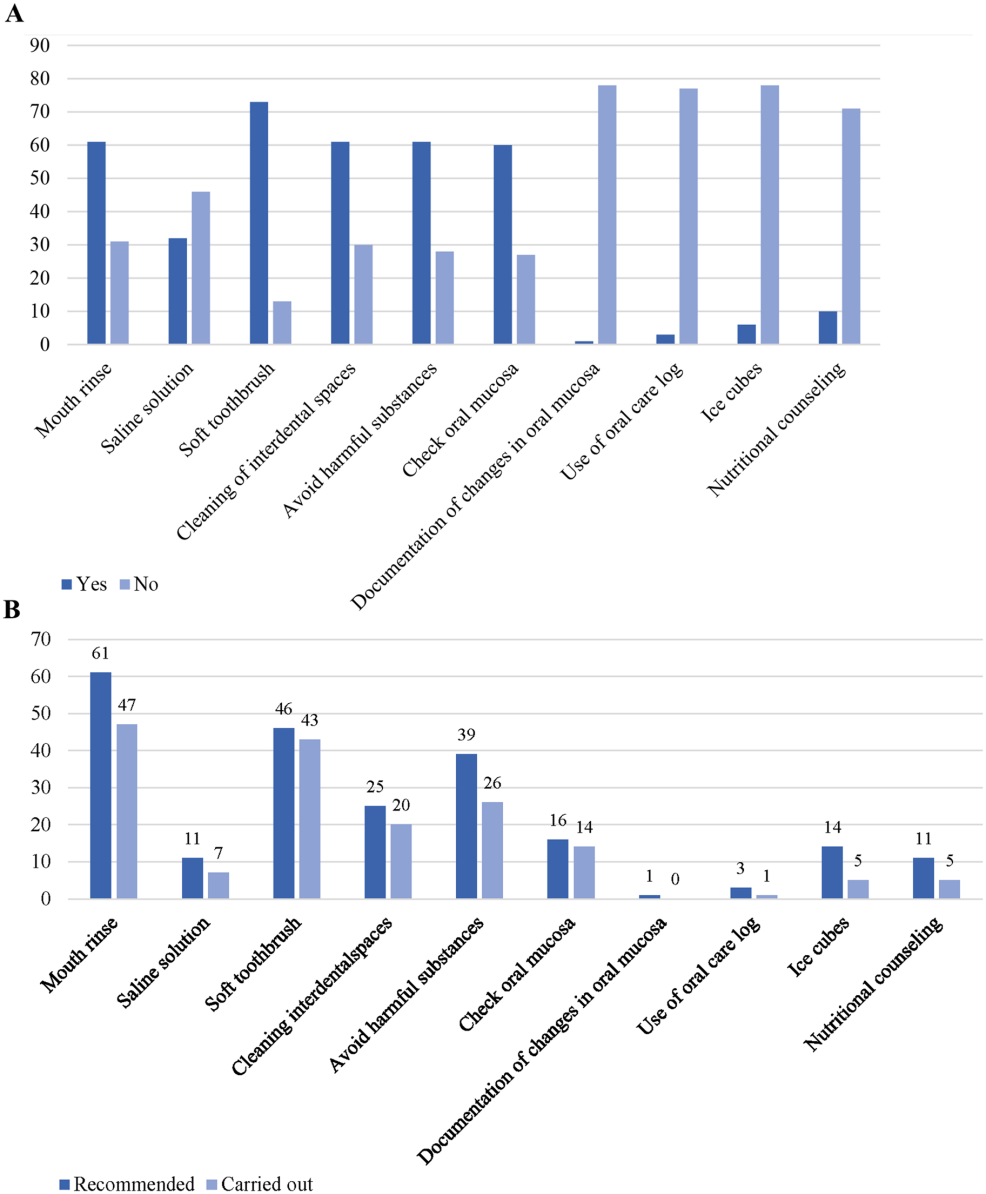
**a** Carried out measures to prevent or treat OM (*N* = 105). **b** Measures recommended by the treating physician and thereof carried out measures (*N* = 86)

We asked patients which prophylactic and therapeutic treatments of the methods listed in the guideline they used for their OM. The method most applied was using a soft toothbrush (59.8%). The proportion of females (70.5%, *n* = 43) abstaining from noxious substances was significantly higher (*p* = 0.012) than the males (27.9%, *n* = 17). (Fig. 2a)

17 of 105 patients (13.9%) stated to use other methods to prevent and/or treat OM besides those listed in the guidelines.

### Methods recommended by the treating physician

The most frequent recommendation from the guideline used by the physician was to rinse the mouth regularly (50%, *n* = 61). 47 (77%) adhered to this recommendation. Nearly all the patients (93.5%, 43 of 46) who declared they got the recommendation to use a soft toothbrush by their treating physician, used it. In general, from the point of view of the patients, they did not receive recommendations for most of the methods listed in the guideline. (Fig. 2b)

Focusing on pain, 58 (47.5%) of the participants stated that they had been informed about the possibility of using a mouthwash with analgesics and 18 (14.8%) about the possibility of taking painkillers.

The patients stated on a total of 303 occasions that they had received recommendations regarding prophylactic and therapeutic measures. They stated 368 times that they had implemented these measures.

Some patients used methods without having been recommended, e.g. using a soft toothbrush (18, 54.5%), cleaning their interdental spaces (24, 52.2%) or checking their oral mucosa regularly (29, 52.7%). (eSupplement Fig e3)

## Discussion

OM poses a frequent side effect of CT/RCT. In this work, we have investigated to what extent the physician notified patients about OM as potential side effect of CT/RCT and the corresponding prophylactic and therapeutic measures.

A majority of patients (61.6%) learned about the risk of OM from their treating physician. Yet, this number is rather low, considering that OM is a major side effect influencing the ability to adhere to healthy eating, thus imposing the risk of malnutrition (Leitlinienprogramm Onkologie (Deutsche Krebsgesellschaft 2020, Allana, Shamsi et al. 2024, Bell and Kasi 2024). A significant proportion of patients (78.3%) learned about OM before or at the start of treatment, indicating proactive patient education A high percentage of patients (89%) declared to have noticed the symptoms of OM before their physician, implying a good sensitization for possible symptoms of OM. A greater part of the patients (63.5%) felt very well informed on treatment options, while a minority (*n* = 14) felt that they were not informed at all. Possible causes could be a different need for information, insufficient time for the conversation or different expectations of the patients. A comparatively low number of patients (17.6%) were recommended to consult a dentist, suggesting that dental referrals for improving oral hygiene and reducing the risk of OM are under-utilized and under-estimated. Several studies have shown a 20–70% reduction in the incidence of OM through professional dental care (Borowski, Benhamou et al. 1994, Soga, Sugiura et al. 2010, Saito, Watanabe et al. 2014, Daugėlaitė, Užkuraitytė et al. 2019). Therefore, interdisciplinary collaboration is important and a dental consultation with specific risk-adapted preventive measures should be firmly integrated into the treatment plan and performed regularly (Elad, Cheng et al. 2020, Leitlinienprogramm Onkologie (Deutsche Krebsgesellschaft 2020). Saito et al. describe dental intervention as a universal component of tumor therapy (Saito, Watanabe et al. 2014). Our results show that this implementation is often insufficient, which is consistent with the observations of Peterson et al., who emphasize the importance of these measures but also criticize their often difficult implementation (Peterson, Bensadoun et al. 2011). However, the low number of referrals to the dentist could be explained by the types of cancer and their CT drugs which did not necessarily require a dental consultation.

### Regarding treatment and prophylaxis options

Only a few patients were recommended or used an oral care protocol even though this is recommended in almost all tumor therapies, including by the MASCC/ISOO clinical practice guidelines (McGuire, Fulton et al. 2013, Lalla, Brennan et al. 2019, Elad, Cheng et al. 2020, Padure, Horhat et al. 2024). Also, only a few patients documented changes in their oral mucosa. This could be due to a lack of information or provision of e.g. a structured questionnaire to simplify documentation. A lack of awareness on the part of patients to document their oral mucosal changes could also be a cause. It’s important to improve awareness of these changes because patients can possibly detect OM earlier and intervention can be made sooner. The use of mouthwashes was the most frequently recommended and used prophylactic measure (50% recommended, 77% used). This could be due to this procedure being easy to use and widespread in our society. The vast majority of patients who were advised to use a soft toothbrush complied with this recommendation (93.5%), showing high compliance with this simple but important measure. Also in total the majority of patients (59.8%) used a soft toothbrush, which is consistent with the recommendations for gentle oral care in OM treatment (Thomson M. et al. 2015, Leitlinienprogramm Onkologie (Deutsche Krebsgesellschaft 2020). New studies have shown that targeted measures can sometimes prevent OM (Cidon 2018, Allana, Shamsi et al. 2024). In particular, it is known that basic measures like mouth rinsing and oral care play an important role here (Epstein, Thariat et al. 2012, Thomson M. et al. 2015, Shankar, Roy et al. 2017, Atwiine, Kyomya et al. 2024, Padure, Horhat et al. 2024). Our results show that patients are already well-informed about these measures. Female patients were more willing to abstain from harmful substances, which indicates gender-specific differences in health behavior. We couldn’t find any studies specifically encouraging women to avoid noxious substances in OM, but studies have shown that women generally consume less harmful substances like tobacco than men (Patel J. et al. 2016, Rehman, Zafar et al. 2018). Rehman et al. also found that more women emphasize dental hygiene (Rehman, Zafar et al. 2018).

Not all measures from the guidelines were recommended to patients by their treating physician. This indicates possible discrepancies between the guideline recommendations and the information provided to patients by their treating physician. The patients reported undertaking more measures than they had been informed about by the physician. The inadequate implementation of guidelines and the communication deficits in the physician-patient relationship are well-known challenges (Peterson et al. 2011; Popa-Velea and Purcărea 2014). Studies also show that patients increasingly wish to be involved in their treatment and the associated decisions (Schipper et al. 2016), which could be reflected in a certain degree of patient initiative.

### Limitations

This study has some relevant limitations. By collecting the data using a questionnaire, a selection bias is possible: Only people who have completed the questionnaire are included in the study (these could be people who are more interested in their health or have a greater need for information). This could lead to the results not being representative. Statements can therefore only be made about the patients who completed the questionnaire. In the survey, relevant patient demographics, such as education status, were not collected. Therefore, it was not possible to analyze their relevance for the outcome. Another limitation of the study is the inclusion of patients whose tumor therapy with the risk of oral mucositis had not yet started. These patients were still included to allow assessment communication and education about oral mucositis even before the start of treatment.

Also, it is noteworthy that it was not feasible to ascertain from the collected data which oncological diseases the patients were afflicted with. Therefore, there may be an uneven distribution. The retrospective, self-reported data collection carries the risk of recall bias. Subgroup analyses by tumor type or treatment method were not performed, which could limit transferability. In addition, no multivariate adjustment was made for possible confounding factors such as age or treatment status. The questionnaire used was not formally validated and information on reliability (e.g. Cronbach’s alpha) is missing. Consequently, the results should be interpreted with caution.

## Conclusion

Overall, these findings highlight the importance of effective communication between patients and clinicians in OM treatment and the need for further research and interventions to improve adherence to guideline recommendations and optimize patient outcomes. It is very important to provide patients with comprehensive and understandable information before or at the start of treatment. Many patients are not fully informed about their disease and the associated risks and do not benefit enough from the available prophylactic and therapeutic options, and these should be addressed more frequently. There is good evidence about the basic prophylactic and therapeutic measures of mouth rinsing and brushing with a soft toothbrush. The patients applied more measures than they were recommended by their physician. This could indicate a certain degree of initiative on the part of the patients.

To improve the situation, patient education and access to relevant information should be optimized. This can be achieved through the distribution of information flyers, training of nursing staff and increased interdisciplinary cooperation. Dental consultations should be firmly integrated into the patient’s treatment plan to ensure holistic care and better prevent OM.

To improve the education and provision of information to patients and thus optimize treatment outcomes, the following measures could be implemented to a greater extent:

*Recommendations for improving patient care*:

1. Create flyers and graphic displays for information materials
2. Review materials together and improve communication
3. Sensitization of nursing staff and caregivers
4. *Improvement of interdisciplinary co-operation*.
5. Regular further education and communication training
6. Guidelines in simple Language and simplified access to information

These recommendations aim to optimize patient care through clear communication, continuous education, and improved interdisciplinary collaboration.

## Electronic supplementary material

Below is the link to the electronic supplementary material.

Supplementary Material 1

## Data Availability

No datasets were generated or analysed during the current study.
