# Supplementary material for: Evaluation of patient management of (radio-)chemotherapy-caused mucositis with the goal of enhancing patient treatment
Source: J Cancer Res Clin Oncol. 2025 Jul 10;151(7):211. doi: 10.1007/s00432-025-06238-2 (PMC12241179; doi:10.1007/s00432-025-06238-2)
Supplement: Supplementary file 1 — Supplementary Material 1 [file 432_2025_6238_MOESM1_ESM.docx]

**-SUPPLEMENT-**

**Assessment of patient care for oral mucositis caused by (radio-) chemotherapy with the aim of improving patient therapy**

Helena Wolff¹, Bijan Zomorodbakhsch^2^**,** Martin Schnizer^1^, Christian Keinki¹, Jutta Hübner¹

Universitätsklinikum Jena, Medizinische Klinik II, Hämatologie und Internistische Onkologie, Am Klinikum 1, 07747 Jena, Friedrich Schiller University Jena, Germany

# ^2^ MVZ Onkologische Kooperation Harz GbR, Innere Medizin, Hämatologie und Internistische Onkologie, Palliativmedizin, Kösliner Straße 14, 38642 Goslar, Germany

**Corresponding author:**

Helena Wolff

Hlobo246@gmx.de

+4915209800998

Jutta Hübner

Jutta.huebner@med.uni-jena.de

**CONTENT**

***eSupplement material e1:*** *standardized questionnaire*

***eSupplement Fig e2****: Dental care methods recommended to the patients by their physicians (N=44)*

***eSupplement Fig e3:*** *Methods by the patients carried out not explicitly stated by the physician (N=71)*

***eSupplement material e1:*** *standardized questionnaire*

**Dear Sir/Madam…**

The majority of patients with malignant tumors receive chemotherapy, i.e. is a drug therapy to prevent the tumor cells from growing further. However, chemotherapy can have many side effects, including inflammation of the oral mucosa. This occurs because, in addition to the tumor cells, the healthy cells of the oral mucosa are damaged as well, making the mucosa less able to regenerate and more sensitive to environmental influences. Oral mucositis can range from simple inflammation, sometimes with sores and redness, to spontaneously bleeding mucosal lesions, and thus can be divided into different degrees of severity.

Some patients are restricted in eating, drinking, swallowing, speaking, and in some cases sleeping due to mild to severe pain.

With the help of this questionnaire, we would like to find out in the context of a scientific investigation, which preventive and therapeutic measures for the improvement of the mouth mucosa inflammation are known to the patients and which you use. In doing so, our goal is to improve patient satisfaction and well-being.

The following questionnaire can be completed by all patients taking tumor medications or also receiving radio-chemotherapy.

We would very much appreciate your participation and would therefore like to invite you to take part in our survey.

Data will be collected anonymously. Consequently, no conclusions can be drawn about your person by participating in the survey. Of course, answering the questions is voluntary and has no influence on your treatment. With your participation, you help us to analyze and improve preventive and therapeutic measures, as well as the associated doctor-patient communication.

In the following questions, the generic masculine is used for simplification and better readability. Female and other gender identities are explicitly included.

Anonymized data is collected, stored and analyzed. The use of data is in accordance with legal requirements and requires your consent.

*Participation requires the following:*

**1.**I agree that anonymous data about my person will be collected during this survey and stored in paper form, as well as on electronic data carriers of password-protected computers for scientific evaluation.

**2.** Furthermore I agree that authorized and to the discretion authorities, who are bound to secrecy, may inspect the anonymized data, as far as this is necessary for the verification of the proper conduct of the study.

**3.** I have been informed that my participation in the survey is voluntary and that I give my consent. The consent to the collection and processing of my anonymous data is irrevocable. Due to the anonymous collection of the data, it can no longer be deleted retroactively.

**4.** I agree that my data will be stored for at least ten years after the end of the survey. After this period my data will be deleted unless this is contrary to legal or statutory retention periods.

By completing and returning the questionnaire, you agree to participate in the survey.

Integrative Oncology Jena led by Prof. Dr. med. Jutta Hübner

1. **Please select your gender with a cross.**

□ male

□ female

□ diverse

1. **Please select your age in years.**

_____years.

1. **What tumor medications are you receiving or have you received?**

□ I get infusions with chemotherapy.

□ I get infusions with other tumor drugs.

□ I get tumor drugs in form of pills.

If you remember the names of the drugs, you can write them down here:______________

□ I am receiving radio-chemotherapy.

□ I don't know.

1. **What is the status of your tumor therapy?**

□ The treatment has not started yet.

□ The treatment is ongoing.

□ The treatment has already been completed.

1. **When did you learn about the possible side effect of oral mucositis from tumor therapy?**

□ I learned about oral mucositis as a side effect before starting therapy.

□ I learned about oral mucositis as a side effect when I started therapy.

□ I learned about oral mucositis as a side effect during therapy while I was not sick with it myself.

□ I learned about oral mucositis as a side effect during therapy while I was sick with it myself.

□ I only learned about oral mucositis as a side effect after the end of therapy because I was not sick with it myself.

1. **Through whom did you learn about the possible side effect of oral mucositis caused by the tumor therapy?**

*(The treating doctor is the doctor who prescribed the tumor medication or supervised the radiation therapy)*

□ I learned about it through my treating doctor.

□ I learned about it from my family doctor.

□ I learned about it from my dentist.

□ I learned about it through the Internet.

□ I learned about it through other people affected by it.

□ I found out about it through friends/acquaintances.

□ I found out about it myself.

1. **There are several risk factors that promote the occurrence of oral mucositis.**

**These include the following:**

*(Please select what applies to you)*

□ I frequently have tartar, bleeding gums, bad breath and/or cavities.

□ I often have a very dry mouth or low salivation.

□ In my family (parents/grandparents/siblings), gingivitis is more common.

□ I am known to have impaired kidney or liver function.

□ I have had tumor therapy before.

□ None of the above applies to me.

1. **There are possible measures for the prevention and therapy of oral mucositis. Which of the following measures have you been informed about by your doctor?**

*(“doctor” refers to the doctor who prescribed the tumor medication or supervised the radiotherapy)*

|  | **Yes** | **No** | **I don´t know** |
| --- | --- | --- | --- |
| **My doctor has advised me to rinse my mouth regularly with a mouth rinse.** |  |  |  |
| **My doctor has recommended water or a saline solution as mouth rinse.** |  |  |  |
| **My doctor has recommended a soft toothbrush for dental care to protect my oral mucosa.** |  |  |  |
| **My doctor has recommended regular cleaning of the interdental spaces with dental floss/interdental brushes.** |  |  |  |
| **My doctor has advised me to avoid harmful substances (e.g. alcohol/sugary drinks, tobacco, spicy & hot foods, acidic foods).** |  |  |  |
| **My doctor has recommended regular examination of my oral mucosa for sores & pain.** |  |  |  |
| **My doctor has recommended that I keep an oral care log to document changes in my oral mucosa.** |  |  |  |
| **My doctor has recommended that I document procedures performed (for example by myself or by my dentist).** |  |  |  |
| **My doctor has informed me about the possibility of cooling the mucous membrane by sucking ice cubes during the infusion of tumor medication.** |  |  |  |
| **My doctor has sent me for nutritional counseling.** |  |  |  |

1. **Did your treating doctor send you to the dentist?**

*(“treating doctor” refers to the doctor who prescribed your tumor medications or supervised your radiation therapy)*

□ Yes

□ No

□ I don´t know.

1. **If you answered yes to the previous question or went to the dentist on your own, what preventive measures were taken by your dentist?**

*(Multiple answers possible)*

□ No measures were carried out.

□ Teeth with caries were treated, for example with fillings.

□ Dead or inflamed teeth were root canal treated or removed.

□ Sharp edges or interference points on teeth and/or dentures were smoothed.

□ Mucosal defects/wounds were treated.

□ Denture pressure points were treated and the denture was repaired.

□ Fluoride was applied to the teeth.

□ A dental cleaning was performed.

□ He gave me precise instructions on oral hygiene.

1. **Who first diagnosed your oral mucositis?**

□ By myself.

□ Through my treating doctor.

□ Through my dentist.

□ Through my caregiver.

□ Other: ______________

□ I don't remember.

1. **How well did you feel informed by your doctor about treatment options?**

*(“doctor” refers to the doctor who prescribed your tumor medication or supervised your radiation therapy).*

□ My doctor recommended therapy options to me and explained them comprehensively.

□ My doctor told me about therapy options but did not explain them further.

□ My doctor did not explain therapy options to me until I asked.

□ My doctor did not explain any therapy options at all.

1. **Did you previously or do you still have any complaints due to the oral mucosa inflammation?**

□ Yes

□ No

***If you selected "Yes" here, please answer the following questions. If you don’t have or never had any symptoms, you can go directly to question 16.***

1. **Thinking back to when the discomfort was most severe, how much did you feel or do you feel limited by the oral mucositis in doing the following?**

*(Please select with a cross where applicable)*

|  | **Strongly restricted** | **Moderately restricted** | **Barely restricted** | **Not restricted at all** |
| --- | --- | --- | --- | --- |
| **I felt when eating:** |  |  |  |  |
| **I felt when drinking:** |  |  |  |  |
| **I felt when swallowing:** |  |  |  |  |
| **I felt when speaking:** |  |  |  |  |
| **I felt when sleeping:** |  |  |  |  |

1. **Did you previously or do you still have pain from the oral mucositis?**

□ Yes

□ No

1. **The following therapeutic measures to alleviate oral mucositis, in addition to those previously mentioned are generally known. Which have you been informed about by your doctor?**

*(“doctor” refers to the doctor who prescribed the tumor medication or supervised the radiation therapy)*

|  | **Yes** | **No** | **I don´t know** |
| --- | --- | --- | --- |
| **I was recommended a mouthwash with pain-relieving properties.** |  |  |  |
| **I was told about the possibility of prescribing and taking painkillers.** |  |  |  |

1. **Have you done anything yourself to treat the oral mucositis? Please select which of the measures you have done or are doing?**

*(Please select the one that applies)*

|  | **Yes** | **No** | **I don´t know** |
| --- | --- | --- | --- |
| **I rinse regularly with a mouth rinse.** |  |  |  |
| **I use water or saline solution as a mouth rinse.** |  |  |  |
| **I use a soft toothbrush to protect my oral mucosa.** |  |  |  |
| **I clean my interdental spaces with dental floss and/or small brushes.** |  |  |  |
| **I avoid harmful substances (for example alcohol/sugar solutions, tobacco, spicy & hot foods, acidic foods).** |  |  |  |
| **I check my oral mucosa regularly for pain and sores.** |  |  |  |
| **I record changes in my oral mucosa, in an oral care log.** |  |  |  |
| **I record measures carried out (by myself or, for example, by my dentist) in an oral care record.** |  |  |  |
| **I cool my oral mucosa during the infusion of tumor medication by sucking ice cubes.** |  |  |  |
| **I have been going to nutritional counseling and following the recommendations.** |  |  |  |

1. **If you have taken any other measures to prevent and/or treat oral mucositis besides those listed in the table above, please list them here:**

***Thank you very much for your support and we wish you all the best on the further road to recovery!***

***eSupplement Fig e1:*** *Dental care methods recommended to the patients by their physicians (N=44)*

***eSupplement Fig e2:*** *Methods by the patients carried out not explicitly stated by the physician (N=71)*
